# Mercury Bioaccumulation and Prediction in Terrestrial Insects from Soil in Huludao City, Northeast China

**DOI:** 10.1007/s00128-012-0649-0

**Published:** 2012-04-22

**Authors:** Zhongsheng Zhang, Xiaolin Song, Qichao Wang, Xianguo Lu

**Affiliations:** Key laboratory of Wetland Ecology and Environment, Institute of Northeast Geography and Agrocology, Chinese Academy of Sciences, Changchun, 130012 China

**Keywords:** Mercury, Bioaccumulation, Insect, Empirical equation

## Abstract

Mercury accumulation was investigated by constructing and testing empirical equations based on mercury in soil (*C*_*s*_) and in 10 terrestrial insects (*C*_*i*_). *C*_*s*_ ranged from 0.13 to 41.01 mg/kg. *C*_*i*_ differed with species and the highest was found in dragonfly. *C*_*s*_ and *C*_*i*_ showed a good linear fit, and a simple equation was used in predicting *C*_*i*_ when insects were classified into one *Insecta* group (*r* = 0.3399, *p* = 0.0037). The taxonomy can affect validities of empirical equations, which fit field data well when insects were grouped by feeding habits, and when grouped by species, empirical equations were suitable only for certain insects.

Mercury is a representative toxic element and has raised significant concern around the world because of its capacity for bioaccumulation in fish and birds (Boening 2001). Once mercury enters the environment, it can be transformed into organic forms such as methylmercury, which can accumulate in the food chain. The biogeochemical cycle of mercury in aquatic ecosystems has been extensively studied over the past several decades (Simoneau et al. 2005; Houserovaá et al. 2007; Liu et al. 2010). Cui et al. (2011) quantified the biomagnification factors of mercury in fish and birds using the stable isotope *δ* ^15^N for trophic level determination in the Yellow River Delta, which is a growing coastal wetland in China. Ackerman et al. (2010) examined mercury bioaccumulation in two aquatic macroinvertebrate taxa in the Central Valley of California. They found high mercury levels in Notonectidae and Corixidae at 1.18 and 0.89 μg/g, respectively. However, relatively fewer studies focused on mercury bioaccumulation in terrestrial insects, although reports on mercury concentrations and accumulation in mammals and birds are widely available (Devkota and Schmidt 2000; Hsu et al. 2006; Heckel and Keener 2007).

Metal concentrations in terrestrial organisms are affected by a number of factors, such as total metal contents in the environment, exposure routes, and organism age (Veltman et al. 2007, 2008; Mierle et al. 2000). Several models, such as the Optimal Modeling for Ecotoxicological Applications (OMEGA), have been used to predict the metal levels in organisms (Cabana and Rasmussen 1994; Heikens et al. 2001; Hendriks and Heikens 2001; Carafa et al. 2009). However, the parameters used in the OMEGA model are complex and difficult to obtain, and most are empirical. Heikens et al. (2001) reported that heavy metals in terrestrial invertebrates can simply be predicted using the metal contents in soil and proved that the model is effective for Zn, Pb, Cu, and Cd. The models used to investigate the transfer of pollutants into organisms usually consider many factors, such as exposure routes, pollutant concentrations, bioavailability, growth dilution, and so on. However, models developed for metal bioaccumulation are often based on data obtained from certain kinds of animals. Thus, the universal applicability of the classificatory scales for mercury remains unclear.

Huludao City is an important nonferrous smelting and chemical industry area in Northeast China. Wuli River and Cishan River are the two main rivers in the city. Over the past few decades, water contaminated with heavy metals has been discharged into these rivers by a chlor-alkali plant and two zinc smelters. Approximately 265 ton mercury was discharged into the Wuli River, of which 95 tons precipitated as sediments while the rest flowed into the Liaodong Basin (Zhao and Yan 1997). Mercury concentrations in water, soil, sediment, and plants near the chlor-alkali and zinc smelters were very high (Zheng et al. 2008). In this study, we determined the amounts of mercury in soil (*C*_*s*_) and insects (*C*_*i*_) to construct and test the model developed by Heikens et al. (2001) using 10 insect species, which were classified according to species, feeding habits, and as one *Insecta* group. This model was used to develop and test empirical equations for the determination of soil mercury accumulation in terrestrial insects, which would be helpful in ecological risk assessments of mercury pollution.

## Materials and Methods

**Fig. 1:**
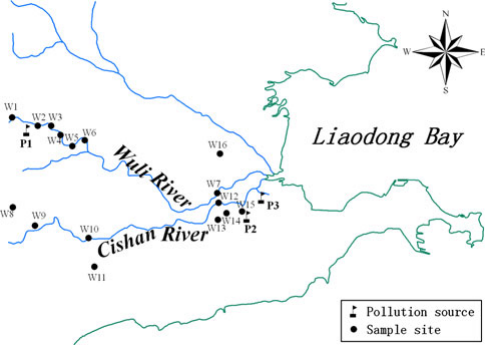
Sample sites (W: sample sites, P: pollution source, P1: the chlor-alkali plant, P2, P3: the zinc smelters)

Familiar terrestrial insects, including grasshoppers (*Locusta migratoria* manilensis and *Acrida chinensis*), spiders, ants, dragonflies, mantises, crickets, *Ambrostoma quadriimpressum* (Motschulsky), cicada, and noctuid larvae were collected manually from 16 sample sites in Huludao City (Fig. 1). Most sites were grasslands near the Wuli River and Cishan River channels. All insects were immediately euthanized with alcohol, preserved in a car-carried refrigerator at 4°C, and brought back to the laboratory. The insects were washed with copious amounts of deionized water to remove surface mercury. The surface moisture on the insects was sucked dry with filters. The insects were then oven-dried at 30°C to obtain a consistent weight, ground into a homogenous powder in a quartz bowl, preserved in polythene bags, and stored in a refrigerator at 4°C prior to use. Soil samples were simultaneously collected with the insects at the same sites. The samples were placed in polyethylene bags, brought back to the laboratory, dried at room temperature, ground and passed through an 80-mesh sieve, and preserved in polyethylene bags prior to use.

The soil and insect samples were all digested using a H_2_SO_4_–HNO_3_–V_2_O_5_ system. Approximately 0.500 g soil or insect sample was mixed with 10.0 mL HNO_3_, 1.0 mL H_2_SO_4_, and 0.030 g V_2_O_5_ and then heated at 130°C. All forms of Hg were converted to Hg^2+^, which was then reduced to elemental Hg by the addition of SnCl_2_. An F-732V Hg detector (Jintan Inc., China) was used to determine the total Hg in soil, and a Tekran 2600 CVAFS (Tekran Inc., Canada) with a detection limit of 5 × 10^−3^ μg/kg was used to determine the total Hg in the insect samples.

The precision and accuracy of the analytical method were evaluated by comparing the expected total Hg concentrations in certified reference materials with the measured values. The expected and measured concentrations in the soil reference (GBW-07405) were 0.290 ± 0.003 and 0.290 ± 0.0015 mg/kg, respectively, whereas the values in the hair reference (GBW-07401) were 0.36 ± 0.05 and 0.39 ± 0.01 mg/kg, respectively. The average recovery rate of mercury was 96.2 %. Simultaneous evaluations of analytical blanks and standard references confirm that the accuracy of the method was within acceptable limits.

All glass equipment were soaked overnight in 3 mol/L HNO_3_, rinsed with copious amounts of distilled deionized water, stored, capped, and filled with deionized water prior to use. All solutions were prepared with distilled deionized water in glass bottles and handled with analytical micropipettes. The reagents used were of excellent pure grades. Statistical analysis was performed using SPSS 10.0 for Windows and Origin 7.5.

## Results and Discussion

**Fig. 2:**
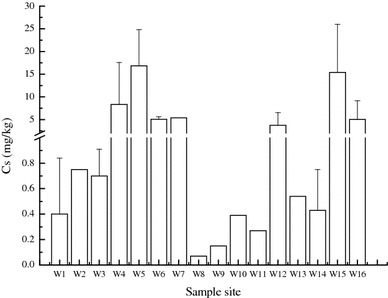
Total mercury in soil of different sample sites

The *C*_*s*_ values obtained were in the 0.13–41.01 mg/kg range, with an average of 5.98 mg/kg. The average value was approximately 160 times greater than the regional mercury background value in the A horizon soil obtained from Liaoning Province. Mercury pollution in Huludao City was serious, and two areas have relatively higher total mercury in soil. These areas are W5–W7 and W15–W16, which are near to the chlor-alkali plant and the zinc smelter (Fig. 2).

Among the 10 insect species collected, *L. migratoria* manilensis, *A. chinensis*, *A. quadriimpressum* (Motschulsky), cicada, and noctuid larvae are herbivores, ants and crickets are omnivores, whereas spiders, dragonflies, and mantises are carnivores (Table 1). *L. migratoria* manilensis and *A. chinensis* are representative arthropods in summer grasslands. Grasshoppers can accumulate mercury from their food, with a bioaccumulation factor of approximately 2.0 (Devkota and Schmidt 2000). In the present study, *C*_*i*_ in *L. migratoria manilensis* and *A. chinensis* were much higher than those reported in literature.

**Table 1:** Summary of total mercury in terrestrial insects

Insect	Range (μg/kg)	Mean (μg/kg)
*Locusta migratoria manilensis*	1.18–621.56	126.15
*Acrida chinensis*	12.82–1,017.67	162.17
*Ambrostoma quadriimpressum* (*Motschulsky*)	7.98–490.07	149.60
Cicada	205–9,987	2,639.18
Noctuid larva	2.32–1,098.65	143.66
Cricket	12.23–865.71	257.61
Ant	57.52–1,206.80	498.32
Dragonfly	232.62–12,432.36	4,234.14
Mantis	180.84–1,321.67	579.10
Spider	14.96–1,098.63	171.94

*C*_*i*_ in cicada was much higher than that in other insects. The high mercury absorption of cicada had been previously reported by Heckel and Keener (2007). This ability may be due to its special living habits. Cicada larvae can live underground for about 4–5 years before their last molting. They suck fluid from plant roots for survival, which can result in the accumulation of high mercury levels because of the relatively high mercury concentrations in plant roots (Boening 2001).

*Ambrostoma quadriimpressum* (Motschulsky) and noctuid larvae feed on plant leaves, and their *C*_*i*_ values were close to each other. ANOVA analysis shows no significant difference in the *C*_*i*_ values of herbivore insect species, except that of cicada.

The order of the *C*_*i*_ values in carnivorous insects is as follows: dragonfly > mantis > spider. *C*_*i*_ in dragonfly was approximately 7 and 25 times higher than those in the mantis and spider, respectively. The mercury concentrations significantly differed among the predator species because of their different special living habits. Dragonfly larvae can live underwater for about 2 years until eclosion. Meanwhile, they prey on zooplankton and little fish as food, resulting in the transfer and accumulation of mercury in their bodies. When dragonflies grow up, they feed on mosquito midges, which have been proven to contain high mercury concentrations (Harding et al. 2006). This feeding habit contributes to the high mercury levels in dragonfly bodies. On the other hand, spiders are one of the predominant hunters of grasshoppers. However, *C*_*i*_ in spider was low and was close to that in grasshopper. Spiders convert the guts of their prey into fluids for food using their venom, which contains digestive enzymes. They do not eat the epicuticle of the prey, which has relatively high metal contents (Heliövaara and Väisänen 1990). In addition, spiders can withstand hunger for several months once they have eaten, and they eat much less than the mantis. Thus, spiders may have lower mercury content in their bodies compared with mantises.

Ants and crickets are omnivorous insects. *C*_*i*_ in ants was higher than that in crickets. Crickets mostly feed on plant leaves, stems, and roots, although they sometimes eat the dead bodies of other insects. Ants eat everything they can find and particularly prefer the dead bodies of other animals, which account for a large proportion of their diet. Thus, the mercury concentrations in ants may be high. In addition, ants live underground, and their skins directly touch the soil. Thus, mercury in soil can enter their bodies through positive or negative diffusion processes.

**Fig. 3:**
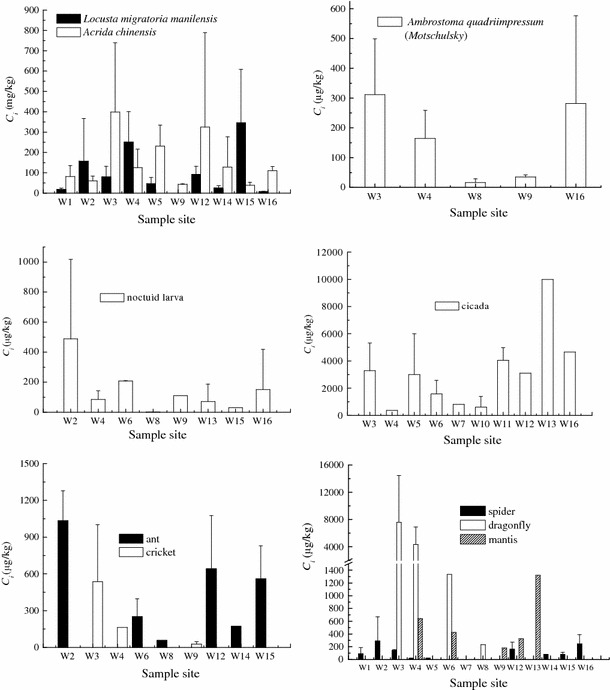
Total mercury concentrations in insects in different sample sites

*C*_*i*_ in sites close to chlor-alkali plant and zinc smelters were obviously higher than those in other sites (Fig. 3). For *L. migratoria manilensis*, *A. quadriimpressum* (Motschulsky), and noctuid larvae, the highest *C*_*i*_ was found in W3, whereas for *A. chinensis* and cicada, the highest *C*_*i*_ was found in site W15. The previous effluent outlet of the chlor-alkali plant was located in W3, and W15 is located just behind the zinc smelter, which explains the increase in the mercury concentrations in the insects in those locations.

Heikens et al. (2001) have reported that the Zn, Pb, Cu, and Cd concentrations in terrestrial invertebrates can be predicted using a simple linear regression equation fitted with the metal concentrations in soil and in the bodies of organisms after a logarithmic transformation. When the insects were grouped by species, the models were valid only for four species, namely, *L. migratoria* manilensis, *A. chinensis*, *A. quadriimpressum* (Motschulsky), and ants (Table 2).

**Table 2:** Regression equations log*C*_*i*_ = log*a* + b*log*C*_*s*_ with the standard deviation (SD)

Insect species	Log*a* ± SD	*b* ± SD	*r*	*p*
*Locusta migratoria manilensis*	0.017 ± 0.398	0.603 ± 0.130	0.868	0.002
*Acrida chinensis*	0.962 ± 0.286	0.363 ± 0.092	0.813	0.004
*Ambrostoma quadriimpressum* (*Motschulsky*)	0.332 ± 0.310	0.525 ± 0.102	0.965	0.036
Ant	−1.653 ± 1.264	2.024 ± 0.522	0.913	0.030

Grasshoppers and ants lay eggs in soil, and their incubation periods are approximately 15–20 days. Afterward, young grasshoppers with limited flying abilities live above the ground, whereas ants live underground and come into contact with the soil all the time. Mercury in soil can penetrate their bodies through absorption or passive diffusion between their skin and soil. This phenomenon may be contributing to the close relationship between the mercury in their bodies and that in soil. The living habits of *A. quadriimpressum* (Motschulsky) do not contribute to mercury accumulation because they often live on trees and feed on elm leaves. Thus, the close correlation between mercury in their bodies and that in soil remains unexplained needs further investigation.

When the insects were groups according to their feeding habits, namely, herbivorous, carnivorous, and omnivorous, a significant linear relationship between *C*_*i*_ and *C*_*s*_ for herbivores and omnivores was observed. The fitted results for the carnivores were not statistically significant (Table 3). The effect of soil on the mercury levels in the herbivorous and omnivorous insects may be due to food intake and skin exposure through the food chain, namely, soil–plant–herbivorous/omnivorous insect–carnivorous insect. Plants absorb mercury from soil. Mercury is then assimilated by the herbivores and omnivores through plant intake. However, carnivores eat meat. Thus, they mainly absorb mercury from their prey, and the effect of soil mercury may be weak. Heikens et al. (2001) reported that when animals were classified according to phylum or subphylum, heavy metals in the bodies of animals were significantly related to metals in soil. However, our results indicate that this prediction may not be applicable for carnivorous animals, which occupy high trophic positions.

**Table 3:** Regression equations with the standard deviation (SD) for insects with different feeding habits

Taxa	Log*a* ± SD	*b* ± SD	*r*	*p*
The herbivorous	0.577 ± 0.283	0.440 ± 0.091	0.6700	<0.0001
The omnivorous	1.018 ± 0.530	0.409 ± 0.165	0.7117	0.048
The carnivorous	1.517 ± 0.529	0.300 ± 0.168	0.3642	0.087

**Fig. 4:**
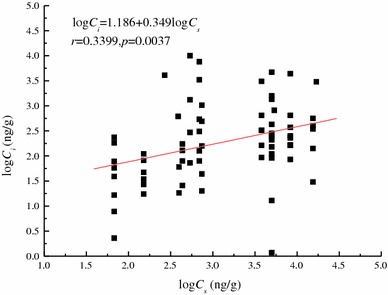
Relationships between *C*_*i*_ and *C*_*s*_ including all insects

When the insects were classified into one *Insecta* group, the linear equation for *C*_*i*_ and *C*_*s*_ fits all biological data well (Fig. 4). The equation indicates that *C*_*i*_ was in good linear regression with *C*_*s*_ after logarithmic transformation. The empirical equation can be used to predict the mercury levels in insects and assess the mercury health risks for the entire insect population at the *Insecta* scale. We tested the equation using the data reported by Hsu et al. (2006).Heavy metal pollution is serious in the industrial zones of Taiwan, and the average total mercury in soil and insects were 0.12 and 0.10 mg/kg, respectively. The predicted value for the total mercury content in insects using the equation was 0.082 mg/kg, which was close to the field data. The result suggests that the equation is useful for ecological risk assessments when insects are grouped at the *Insecta* scale.

In summary, the formulated equations showed a good fit with field data when the insects were classified as one *Insecta* group. When the insects were grouped according to their feeding habits, the model showed a good fit for the herbivores and omnivores, but not for the carnivores. When the insects were grouped according to species, the model showed a good fit for only 4 of 10 species. The validity of the models for predicting the pollutant content in insects was acceptable for large taxonomical groups, such as a class or a phylum. When the insects were further grouped according to feeding habits or species, the accuracy of the models were significantly reduced. Although numerous models and empirical equations were developed to study the metal biomagnifications in food chains, taxonomy should be considered when predicting the pollutant levels in certain kinds of animals using the proposed models.
